# Age Differences in the Association of Sleep Duration Trajectory With Cancer Risk and Cancer-Specific Mortality: Prospective Cohort Study

**DOI:** 10.2196/50836

**Published:** 2024-02-07

**Authors:** Chenan Liu, Qingsong Zhang, Chenning Liu, Tong Liu, Mengmeng Song, Qi Zhang, Hailun Xie, Shiqi Lin, Jiangshan Ren, Yue Chen, Xin Zheng, Jinyu Shi, Li Deng, Hanping Shi, Shouling Wu

**Affiliations:** 1 Department of Gastrointestinal Surgery and Clinical Nutrition Beijing Shijitan Hospital Beijing China; 2 National Clinical Research Center for Geriatric Diseases Xuanwu Hospital Capital Medical University Beijing China; 3 Key Laboratory of of Cancer Food for Special Medical Purposes for State Market Regulation Beijing China; 4 Laboratory for Clinical Medicine Capital Medical University Beijing China; 5 Department of General Surgery Kailuan General Hospital Tangshan China; 6 Department of Obstetrics and Gynecology Dongguan Maternal and Child Health Care Hospital Dongguan China; 7 Cardiovascular Research Institute University of California San Francisco, CA United States; 8 Department of Genetics Yale University School of Medicine New Haven, CT United States; 9 Department of Oncology First Hospital of Shanxi Medical University Taiyuan China; 10 Department of Cardiology Kailuan General Hospital Tangshan China

**Keywords:** sleep duration, aging, cancer risk, mortality, sleep, trajectory, adult

## Abstract

**Background:**

Baseline sleep duration is associated with cancer risk and cancer-specific mortality; however, the association between longitudinal patterns of sleep duration and these risks remains unknown.

**Objective:**

This study aimed to elucidate the association between sleep duration trajectory and cancer risk and cancer-specific mortality.

**Methods:**

The participants recruited in this study were from the Kailuan cohort, with all participants aged between 18 and 98 years and without cancer at baseline. The sleep duration of participants was continuously recorded in 2006, 2008, and 2010. Latent mixture modeling was used to identify shared sleep duration trajectories. Furthermore, the Cox proportional risk model was used to examine the association of sleep duration trajectory with cancer risk and cancer-specific mortality.

**Results:**

A total of 53,273 participants were included in the present study, of whom 40,909 (76.79%) were men and 12,364 (23.21%) were women. The average age of the participants was 49.03 (SD 11.76) years. During a median follow-up of 10.99 (IQR 10.27-11.15) years, 2705 participants developed cancers. Three sleep duration trajectories were identified: normal-stable (44,844/53,273, 84.18%), median-stable (5877/53,273, 11.03%), and decreasing low-stable (2552/53,273, 4.79%). Compared with the normal-stable group, the decreasing low-stable group had increased cancer risk (hazard ratio [HR] 1.39, 95% CI 1.16-1.65) and cancer-specific mortality (HR 1.54, 95% CI 1.18-2.06). Dividing the participants by an age cutoff of 45 years revealed an increase in cancer risk (HR 1.88, 95% CI 1.30-2.71) and cancer-specific mortality (HR 2.52, 95% CI 1.22-5.19) only in participants younger than 45 years, rather than middle-aged or older participants. Joint analysis revealed that compared with participants who had a stable sleep duration within the normal range and did not snore, those with a shortened sleep duration and snoring had the highest cancer risk (HR 2.62, 95% CI 1.46-4.70).

**Conclusions:**

Sleep duration trajectories and quality are closely associated with cancer risk and cancer-specific mortality. However, these associations differ with age and are more pronounced in individuals aged <45 years.

**Trial Registration:**

Chinese Clinical Trial Registry ChiCTR–TNRC–11001489; http://tinyurl.com/2u89hrhx

## Introduction

Sleep, a body and brain repair agent, plays a vital role in development, growth, and metabolism [1]. Regular and high-quality sleep can promote brain waste clearance, memory recovery, immune health, and normal nutrient metabolism. Correspondingly, sleep disorders may seriously affect the health and quality of life of patients [2].

Unfortunately, sleep disorders are one of the most common issues in today’s society. The World Health Organization has reported that approximately 27% of the population experiences sleep disorders. Therefore, scientists have conducted extensive studies on sleep disorders, most of which have focused on rapid eye movement (REM) sleep behavior disorders [3] or sleep disorders caused by diseases such as obstructive sleep apnea (OSA) [4]. Recently, an authoritative study has identified sleep duration as 1 of the 6 key elements of sleep health [5]. Sleep duration is associated with the occurrence and prognosis of various diseases. Dong et al [6] reported that insufficient or excessive sleep duration can increase the risk of depression. In terms of metabolic health, decreased sleep duration is associated with a higher risk of and a higher severity score for metabolic syndrome [7]. Dynamic changes in sleep duration can also bring significant changes to the body. In experimental research, sleep-deprived mice experience disruptions in their blood sugar homeostasis, which is restored to normal when sleep is regained. Cohort studies have also confirmed this point [8]. A study on shift work showed a strong correlation between irregular sleep duration and type 2 diabetes, obesity, heart disease, and cancer [9]. Nevertheless, in most of these studies, the sleep duration of participants was assessed at a single time to determine their cancer risk or even mortality, which is biased. To the best of our knowledge, various factors affect sleep, including the external environment (sound, light, and air quality), depression, anxiety, and age [10]. When considering age, approximately 50% of the older population experiences sleep disorders. Changes in sleep patterns and circadian dysrhythmia are considered part of aging [11]. Sleep duration and quality significantly decrease with age. Surprisingly, compared with young people, lack of sleep in older people has less effect on performance [12]. Therefore, it is likely that a single sleep survey will estimate the risk of disease and death erroneously. For the first time, we measured the sleep duration trajectory by monitoring the sleep of participants from 2006 to 2010 and then elucidated the association of sleep duration trajectories with cancer risk and cancer-specific mortality.

## Methods

### Study Participants

**Figure 1:**
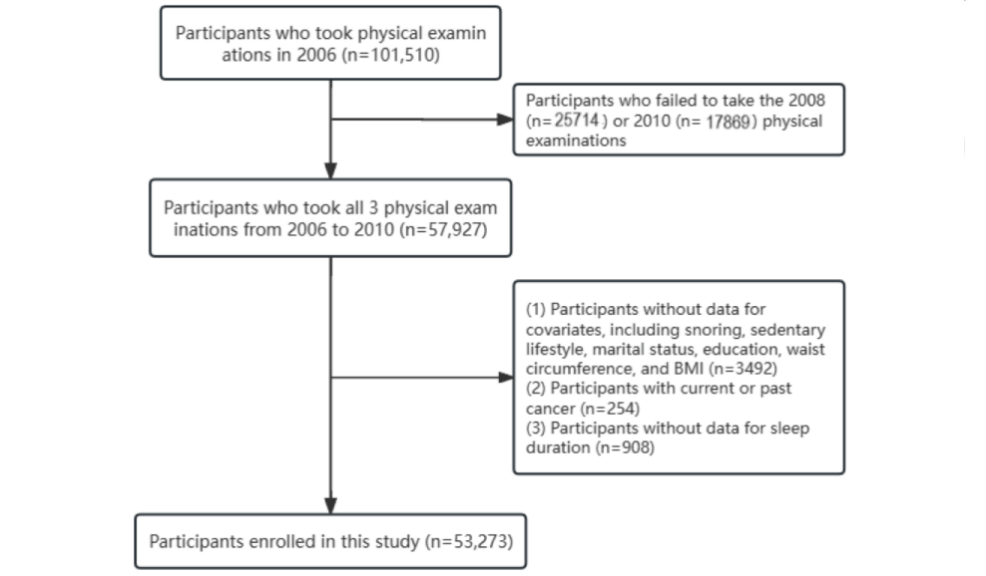
Flowchart of study design.

All participants were from the Kailuan cohort, part of an ongoing prospective study in Tangshan, China (ChiCTR-TNRC-11001489, registered at the Chinese Clinical Trial Registry). As previously described [13], the Kailuan cohort was initiated in 2006, when Kailuan General Hospital collaborated with 11 other hospitals to conduct physical examinations on 101,510 participants; the examinations included clinical examinations, health questionnaires, imaging, and laboratory tests. Thereafter, participants were followed up and periodically resurveyed every 2 years, and relevant indicators were recorded [14]. In this study, to construct the sleep duration trajectory, we included individuals who consecutively participated in the physical examinations from 2006 to 2010 (n=57,927); 3492 participants with missing covariates such as snoring, sedentary lifestyle, marital status, education, waist circumference (WC), BMI, and other covariates were excluded. Furthermore, 254 participants with cancer or cancer at baseline and 908 participants with missing sleep duration information were excluded. Finally, 53,273 participants were included in this study (Figure 1).

### Ethical Considerations

This study was conducted according to the principles of the Declaration of Helsinki and its revised version and was approved by the ethics committee of Kailuan General Hospital with the code sjtkyll-lx-2021(39). All participants agreed to participate in the study and provided written informed consent.

### Exposure and Covariates

All participants were invited to the hospital to fill out the Pittsburgh Sleep Quality Index (PSQI) sleep questionnaire survey (Multimedia Appendix 1) [15]. They reported their sleep status over the past 6 months. Information about the participants’ average sleep duration (3-15 h, excluding naps) was extracted from the questionnaire as exposure. For participants who reported approximate sleep duration, the average value was calculated (for example, 6-7 h was considered 6.5 h). The covariates were age, sex, BMI, WC, marital status, education level, sedentary time, physical activity, smoking, drinking, hypertension, diabetes, snoring, salt intake, family history of tumors, high-sensitivity c-reactive protein (CRP), total cholesterol (TC), triglycerides (TG), and digestive system cancer–related factors (fatty liver, gallstones, cirrhosis, and hepatitis B virus infection). Multimedia Appendix 1, Table S1 presents the definitions of all covariates.

### Outcome Assessment

The electronic medical records from provincial vital statistics offices, the Tangshan medical insurance system, and the Kailuan Social Security Information System were used to determine the time of tumor occurrence and death of participants as well as specific causes of death. Cancer was diagnosed via pathology or imaging. All diagnoses were recorded using the International Classification of Diseases, 10th revision (Multimedia Appendix 1). The follow-up time was from the date of completion of the investigation in 2010 until the occurrence of cancer, death, or the last follow-up date (December 31, 2021), whichever came first.

### Statistical Analyses

Statistical analyses were conducted using SAS (version 9.4; SAS Institute) or R (version 4.2.0; R Foundation for Statistical Computing). All *P* values were 2-sided, and a *P* value of <.05 was considered statistically significant.

Self-reported sleep duration trajectories were constructed for participants from 2006 to 2010 using latent mixed modeling, which was performed using Proc Traj in SAS. The construction of the trajectories was gradual. First, we established 5 types of trajectories and then compared them with 4, 3, 2, and 1 trajectories. The Bayesian information criterion was used to evaluate the fit of each trajectory model. Furthermore, models with different functional forms were evaluated based on the significance levels of cubic, quadratic, and linear terms. Continuous variables with normal distributions were expressed as mean (SD) and compared between groups using a 1-way ANOVA. Data with skewed distributions were expressed as median (IQR) and compared using the Kruskal-Wallis test. Categorical variables were expressed as n (%); between-group comparisons were made using the chi-square test. At the same time, Bonferroni correction was used for multiple comparison corrections. After satisfying the proportional hazard ratio assumption, Cox proportional hazards regression models were used to describe the association of different trajectories with cancer risk and cancer-specific mortality. In the adjusted model, the covariates were adjusted. Furthermore, the effect of some potential factors on the risk of digestive system cancers was considered [14,16,17]. Therefore, we additionally adjusted for liver cirrhosis, hepatitis B, gallstones, and gallbladder polyps for digestive system cancers. Because significant differences were observed in sleep patterns and effects between young people and middle-aged and older people [18], we first divided the participants into young people (aged <45 years) and middle-aged and older people (aged ≥45 years) to conduct the study. After observing that sleep duration was more closely related in young people, we conducted more detailed analyses, including subgroup and sensitivity analyses, in participants aged <45 years. For subgroup analysis, previously reported potential modifiers, such as snoring, sex [19], BMI [20], and regular physical exercise [21], were selected. Simultaneously, to clarify the joint effect of sleep quality and sleep duration trajectory, snoring and sleep duration trajectory were combined and the participants were regrouped. In sensitivity analyses, participants who developed cancer within 1 year of follow-up, those with hepatitis B infection, those with regular physical activity, those with a family history of tumor, and those who used sleep medication at least once during the past month were excluded. Furthermore, the variables were adjusted for other components in the PSQI. To avoid bias owing to temporal changes, we also adjusted for time-varying covariates. Finally, to avoid overestimation of cancer risk by death as a competing event, we repeated the analysis using a competing risk model (Multimedia Appendix 1).

## Results

### Baseline Characteristics

**Figure 2:**
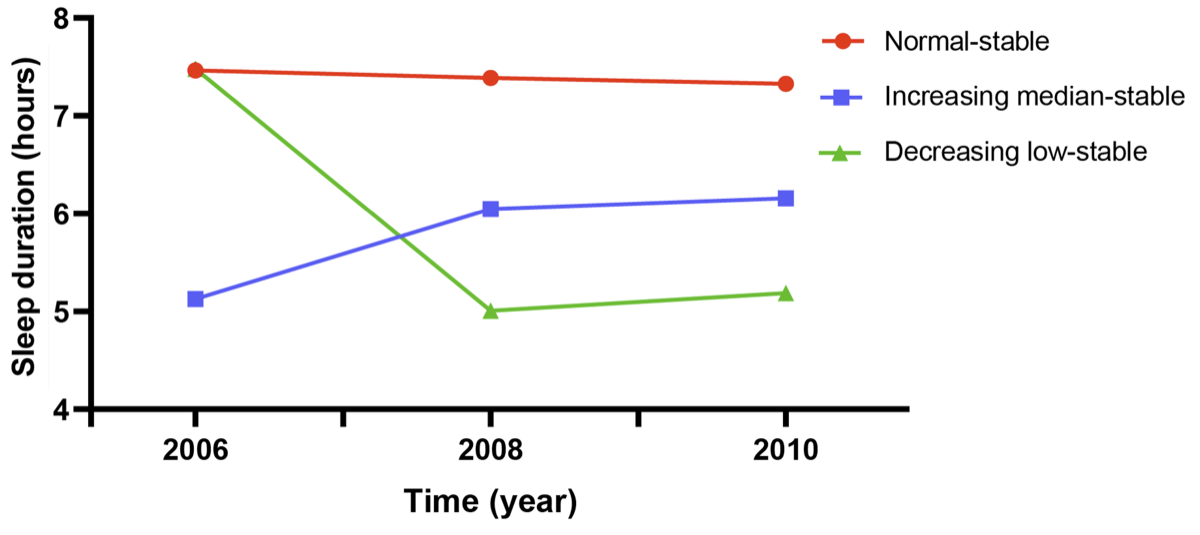
Sleep duration trajectory patterns from 2006 to 2010.

A total of 53,273 participants were included in this study, of whom 40,909 (76.79%) were men and 12,364 (23.21%) were women. The average age of the participants was 49.03 (SD 11.76) years. During a median follow-up of 10.99 years, 2705 participants developed cancers (Multimedia Appendix 1, Table S2); 518 of them were younger than 45 years (Multimedia Appendix 1, Table S3) and 2187 were aged 45 years or older (Multimedia Appendix 1, Table S4). According to the sleep duration of participants from 2006 to 2010, we constructed 3 trajectory patterns. A total of 84.18% (44,844/53,273) of the participants were categorized into the normal-stable group (mean sleep duration range 7.32-7.46 h), 11.03% (5877/53,273) into the increasing median-stable group (mean sleep duration range 5.23-6.09 h), and 4.79% (2552/53,273) into the decreasing low-stable group (mean sleep duration range 5.01-7.48 h) (Figure 2). Among the participants overall (Table 1) and those younger than 45 years (Multimedia Appendix 1, Table S5), the increasing median-stable and decreasing low-stable groups were older, had higher WC and TC, and were more likely to drink, snore, and have fatty liver than the normal-stable group, but had similar BMI, CRP, and TG levels. However, among middle-aged and older participants (Multimedia Appendix 1, Table S6), those in the increasing median-stable and decreasing low-stable groups were older; had higher TC; were more likely to have a sedentary lifestyle, snore, have fatty liver, and consume a high-salt diet; and had lower TG, WC, and BMI compared with participants in the normal group. The Bonferroni correction indicated that when conducting pairwise comparisons among the 3 groups, there were differences in age, education, and alcohol use (Multimedia Appendix 1, Table S7).

**Table 1:** Basic characteristics of the participants by sleep duration trajectory.

Characteristics			Normal-stable (n=44,844)		Increasing median-stable (n=5877)		Decreasing low-stable (n=2552)		*P* value
Age (years), mean (SD)			48.51 (11.8)		52.04 (11.4)		51.32 (11.1)		<.001
**Age (years), n (%)**									
	<45	17,674 (39.4)		1577 (26.8)		698 (27.4)		<.001	
	≥45	27,170 (60.6)		4300 (73.2)		1854 (72.6)			
Male sex, n (%)			34,323 (76.5)		4676 (79.6)		1910 (74.8)		<.001
**BMI, n (%)**									
	<18.5 kg/m^2^	17,555 (39.1)		2261 (38.5)		989 (38.8)		.13	
	18.5-23.9 kg/m^2^	18,739 (41.8)		2552 (43.4)		1090 (42.7)			
	≥24 kg/m^2^	8550 (19.1)		1064 (18.1)		473 (18.5)			
BMI (kg/m^2^), mean (SD)			25.11 (3.5)		25.08 (3.4)		25.05 (3.4)		.61
Waist circumference (cm), median (IQR)			86.0 (80.0-93.0)		86.5 (80.0-93.0)		87.0 (81.0-93.0)		<.001
Married, n (%)			42,658 (95.1)		5479 (93.2)		2439 (95.6)		<.001
College graduate or above, n (%)			10,175 (22.7)		1574 (26.8)		496 (19.4)		<.001
Sedentary time ≥8 hours, n (%)			10,831 (24.2)		2205 (37.5)		606 (23.7)		<.001
Regular physical activity, n (%)			6027 (13.4)		1380 (23.5)		379 (14.9)		<.001
Smoker, n (%)			13,254 (29.6)		2527 (43)		734 (28.8)		<.001
Uses alcohol, n (%)			7479 (16.7)		1663 (28.3)		486 (19)		<.001
Hypertension, n (%)			17,615 (39.3)		2395 (40.8)		992 (38.9)		.08
Diabetes mellitus, n (%)			3570 (8)		502 (8.5)		226 (8.9)		.10
Snoring, n (%)			15,838 (35.3)		3412 (58.1)		950 (37.2)		<.001
**Salt consumption, n (%)**									
	<6 g/day	4047 (9)		760 (12.9)		197 (7.7)		<.001	
	6-10 g/day	36,351 (81.1)		4050 (69)		2100 (82.3)			
	>10 g/day	4433 (9.9)		1063 (18.1)		255 (10)			
Family history of tumor, n (%)			1722 (3.8)		437 (7.4)		90 (3.5)		<.001
C-reactive protein (mg/L), mean (SD)			2.16 (5.30)		2.17 (7.31)		2.32 (5.83)		.39
Total cholesterol (mmol/L), median (IQR)			4.9 (4.3-5.6)		5.0 (4.3-5.7)		5.0 (4.3-5.7)		<.001
Triglyceride (mmol/L), median (IQR)			1.3 (0.9-1.9)		1.28 (0.9-2.0)		1.3 (0.9-1.9)		.55
Fatty liver, n (%)			14,512 (32.4)		2197 (37.4)		907 (35.5)		<.001
Gallstone disease, n (%)			978 (2.2)		146 (2.5)		59 (2.3)		.32
Cirrhosis, n (%)			337 (0.8)		47 (0.8)		24 (0.9)		.54
Hepatitis B virus infection, n (%)			1212 (2.7)		151 (2.6)		74 (2.9)		.68

### Association Between Sleep Duration Trajectory and Cancer Risk

For all participants, no significant change in cancer risk was observed in the increasing median-stable group (HR 0.97, 95% CI 0.84-1.11); however, in the decreasing low-stable group, overall cancer risk for the participants increased significantly, by 39% (HR 1.39, 95% CI 1.16-1.65). Interestingly, after dividing the participants based on the age criterion (45 years), this trend was only present among young people (HR 1.88, 95% CI 1.30-2.71) rather than middle-aged or older participants (HR 1.17, 95% CI 0.96-1.42). Among participants younger than 45 years, those in the decreasing low-stable group were at an overall increased risk of cancer compared with those in the normal-stable group (HR 1.88, 95% CI 1.30-2.71) (Table 2). Among cancer types, participants in the decreasing low-stable group only showed an increased risk of lung cancer (HR 1.51, 95% CI 1.08-2.12), pancreatic cancer (HR, 3.08, 95% CI 1.05-9.08), and stomach cancer (HR 2.06, 95% CI 1.10-3.87) (Multimedia Appendix 1, Table S8). Similarly, the risk of lung cancer (HR 3.64, 95% CI 1.64-8.09), esophageal cancer (HR 4.92, 95% CI 1.08-12.16), pancreatic cancer (HR 7.16, 95% CI 1.83-31.62), liver cancer (HR 3.68, 95% CI 1.04-8.03), and digestive system cancer (HR 2.43, 95% CI 1.23-4.81) increased among participants younger than 45 years in the decreasing low-stable group and not among the middle-aged or older participants (Multimedia Appendix 1, Figure S1).

**Table 2:** Hazard ratios (HRs) for the association of sleep duration trajectory patterns with overall cancer risk.

Sleep duration trajectory patterns		Cases/total	IR^a^	Crude model HR (95% CI)	*P* value	Adjusted model^b^ HR (95% CI)	*P* value
**Overall**							
	Normal-stable	2235/44,844	4.80	Reference		Reference	
	Increasing median-stable	299/5877	4.93	1.03 (0.91-1.16)	.67	0.97 (0.84-1.11)	.62
	Decreasing low-stable	171/2552	6.57	1.37 (1.17-1.60)	<.001	1.39 (1.16-1.65)	<.001
**Age <45 years**							
	Normal-stable	437/17,674	2.28	Reference		Reference	
	Increasing median-stable	35/1577	2.03	0.89 (0.69-1.25)	.50	0.89 (0.59-1.36)	.75
	Decreasing low-stable	31/698	4.12	1.81 (1.25-2.60)	.001	1.88 (1.30-2.71)	<.001
**Age ≥45 years**							
	Normal-stable	1798/27,170	6.56	Reference		Reference	
	Increasing median-stable	264/4300	6.08	0.93 (0.81-1.05)	.24	0.83 (0.71-1.02)	.10
	Decreasing low-stable	140/1854	7.57	1.15 (0.97-1.37)	.11	1.17 (0.96-1.42)	.12

^a^IR: incidence rate per 1000 person-years.

^b^The model was adjusted for continuous (age, BMI, waist circumference, c-reactive protein, total cholesterol, triglycerides, and sleep duration in 2010) and categorical (sex, marital status, education level, sedentary time, physical activity, smoking, alcohol use, hypertension, diabetes, snoring, salt intake, and family history of tumor) variables.

### Association Between Sleep Duration Trajectory and Cancer-Specific Mortality

Similar to the findings on sleep duration trajectories and cancer risk, for all participants, compared to the normal-stable group, individuals in the decreasing low-stable group had a significantly higher risk of cancer-specific mortality (HR 1.54, 95% CI 1.18-2.06). The same phenomenon was also observed among participants aged <45 years, where individuals in the decreasing low-stable group had a significantly higher risk of cancer-specific mortality (HR 2.52, 95% CI 1.22-5.19). However, no such association was found among participants aged ≥45 years (HR 1.28, 95% CI 0.94-1.75) (Table 3).

**Table 3:** Hazard ratios (HRs) for the association of sleep duration trajectory patterns with cancer-specific mortality.

Sleep duration trajectory patterns			Cases/total		MR^a^		Cancer-specific HR (95% CI)^b^		*P* value
**Total**									
	Normal-stable	777/44,844		1.64		Ref.			
	Increasing median-stable	115/5877		1.86		1.07 (0.86-1.33)		.71	
	Decreasing low-stable	65/2552		2.44		1.54 (1.18-2.06)		.01	
**Age <45 years**									
	Normal-stable	108/17,674		0.56		Ref.			
	Increasing median-stable	9/1577		0.52		0.80 (0.39-1.65)		.55	
	Decreasing low-stable	11/698		1.44		2.52 (1.22-5.19)		.01	
**Age ≥45 years**									
	Normal-stable	669/27,170		2.39		Ref.			
	Increasing median-stable	106/4300		2.39		1.01 (0.80-1.27)		.96	
	Decreasing low-stable	54/1854		2.84		1.28 (0.94-1.75)		.12	

^a^MR: mortality rate per 1000 person-years.

^b^The model was adjusted for continuous (age, BMI, waist circumference, c-reactive protein, total cholesterol, triglycerides, and sleep duration in 2010) and categorical (sex, marital status, education level, sedentary time, physical activity, smoking, alcohol use, hypertension, diabetes, snoring, salt intake, and family history of tumor) variables.

### Subgroup Analysis and Joint Analysis

We did not find any interactions between sleep duration trajectory and snoring (*P* for interaction .16), sex (*P* for interaction .80), overweight (*P* for interaction .50), and regular physical activity (*P* for interaction .90) (Multimedia Appendix 1, Table S9) in the subgroup analysis; however, in the joint analysis (Multimedia Appendix 1, Table S10), participants in the decreasing low-stable group were at the highest risk of cancer, especially those who snored (HR 2.62, 95% CI 1.46-4.70).

### Additional Analyses

The competing risk model showed that after considering death as a competitive event, a closer association between sleep duration trajectory and cancer risk was observed. In both the cause-specific hazard functions (CSHF) model and the subdistribution hazard functions (SDHF) model, when compared to the normal stable group, participants in the decreasing low-stable group had an elevated risk of lung cancer (CSHF HR 2.42, 95% CI 1.07-5.53; SDHF HR 2.23, 95% CI 1.17-5.25), esophageal cancer (CSHF HR 4.07, 95% CI 1.12-14.89; SDHF HR 4.05, 95% CI 1.13-15.03), pancreatic cancer (CSHF HR 7, 95% CI 1.40-42.319; SDHF HR 6.88, 95% CI 1.38-27.6), liver cancer (CSHF HR 3.37, 95% CI 1.19-9.66; SDHF HR 3.08, 95% CI 1.08-10.40), digestive system cancers (CSHF HR 1.93, 95% CI 1.05-3.62; SDHF HR 1.99, 95% CI 1.23-5.53), and overall cancer risk (CSHF HR 2.01, 95% CI 1.39-3.17; SDHF HR 1.97, 95% CI 1.12-3.08) (Multimedia Appendix 1, Table S11). Finally, after performing multiple sensitivity analyses and adjusting for time-varying covariates, the results remained robust. Compared to the normal-stable group, participants in the decreasing low-stable group still had an increased risk of lung cancer, esophageal cancer, pancreatic cancer, liver cancer, digestive system cancers, and overall cancer risk (Multimedia Appendix 1, Table S12).

## Discussion

### Principal Findings

To the best of our knowledge, this is the first prospective study investigating age-related differences in the association of sleep duration trajectories with cancer risk and cancer-specific mortality. Compared with middle-aged and older individuals, young people in this study showed a closer association between sleep duration trajectories and the aforementioned risks. Despite some participants having baseline sleep durations within the normal range, once their sleep duration decreased, the aforementioned risks increased accordingly. Additionally, individuals with decreasing low-stable and normal-stable sleep duration trajectory and snoring had the highest risk of cancer.

Sleep duration is the most intuitive and concise indicator for evaluating sleep quality. A Mendelian randomization study from the UK Biobank showed that shorter sleep duration was associated with a higher risk of digestive tract cancers, such as stomach and pancreatic cancers [22], and most studies are in agreement with this study. In terms of cancer-related deaths, previous meta-analyses showed that compared with individuals who sleep for 7 to 8 hours, those who sleep for 4 to 5 hours were at an increased risk of cancer mortality [9]. Li et al [23] found an association between short sleep time and a 24% increase in lung cancer mortality. However, there is no consensus due to the existence of contradictory results. A multicenter study from Japan showed that compared with 7 hours of sleep, less than 5 hours of sleep did not seem to affect the incidence rate or mortality of cancer [24]. Several meta-analyses also showed no relationship between sleep duration and breast cancer or overall cancer risk [25,26].

We speculate that the heterogeneity in these results may be because of population-specific factors. Svensson et al [27] conducted one of the largest studies in Asia on the association between sleep duration and mortality, and the results indicated that age, an important modifier, greatly affected the association between sleep duration and prognosis. Another large prospective study was consistent with the abovementioned study, showing that shortened sleep duration increased the mortality rate in individuals younger than 65 years but not in those aged 65 years or older, and the effect of sleep duration on mortality was highest among younger individuals and negatively correlated with age [28]. These results are interesting and enlightening. We initially found an association between sleep duration trajectory and overall cancer risk in the entire population; however, the results differed significantly when the participants were stratified by age. Sleep duration changes only affected the cancer risk and mortality of participants younger than 45 years.

We hypothesize that this outcome is because of age-related differences in sleep patterns, because aging itself is probably accompanied by advanced sleep timing, shortened nocturnal sleep duration, and increased awakenings [29]. Therefore, changes in nocturnal sleep duration may have a lesser effect on older adults. Conversely, significant fluctuations in the sleep duration of young individuals may imply underlying issues in their physiological functioning. Mechanistically, regarding the central nervous system, as individuals age, neurotoxic substances accumulate, leading to cortical thinning and brain white-matter degeneration; additionally, the age-related dysregulation of neurotransmitters, including serotonin and adenosine, which are involved in sleep regulation, impairs the central sleep regulatory system [30]. Considering hormones and circadian rhythms, aging is closely related to a decrease in sex hormone levels. Studies have shown a significant correlation between decreased testosterone levels and shortened sleep duration and increased sleep fragmentation in older adults [31]. Cortisol, a hormone related to circadian rhythms, regulates sleep, and older adults often exhibit abnormalities in their cortisol rhythm, particularly an earlier peak in nocturnal cortisol levels, which leads to increased nocturnal awakenings and decreased slow-wave sleep [29]. The level of melatonin, an essential hormone that regulates circadian rhythms, differs depending on age, showing significantly reduced peak levels in older adults in comparison with younger individuals [32]. Therefore, fluctuations and changes in nocturnal sleep duration in older adults do not necessarily imply any disease but may simply be a normal aging-related progression. However, some studies have shown that changes in sleep patterns in older adults may increase the risk of lung cancer to some extent [33]. Nonetheless, these results do not indicate that sleep is unimportant in middle-aged and older adults. We can only cautiously conclude that compared with younger individuals, relying solely on sleep duration to assess the prognosis of middle-aged and older adults may lack sensitivity, and a comprehensive evaluation of their sleep patterns is crucial.

Compared with age, a factor that was associated with contradictory results, sleep quality is a critical factor. Zhang et al [19] reported similar results by conducting a 22-year prospective cohort study, finding that the effect of changes in sleep duration on colon cancer risk only existed in individuals who snored. Additionally, snoring can also increase the risk of physical weakness. A Chinese study showed that for participants with prefrailty, unhealthy sleep patterns such as snoring increased the risk of frailty by 42% [34]. In attempting to explore the underlying mechanisms, one study entered our field of view [35], which found that snoring or OSA can exacerbate intermittent hypoxia in mouse models and activate the key oxidative stress factor Bach1 in the body, thereby promoting the proliferation, invasion, and migration of lung cancer cells. Simultaneously, these factors promote the stemness of cancer cells and increase the degree of malignancy.

This study has the following limitations. First, the Kailuan cohort is a male-dominated cohort in northern China, which may not represent a general population because sleep differs across races and sexes [36]. Second, when evaluating sleep quality, we only included snoring, whereas REM sleep behavior disorder, restless leg syndrome, and OSA were not included in this study. Third, the information regarding sleep duration was obtained through questionnaires and participants’ recall; thus, the results may be affected by recall bias.

### Conclusions

In conclusion, sleep duration trajectories are closely associated with the risk of cancers, as well as cancer-specific mortality. However, these associations vary with age and are more pronounced in individuals younger than 45 years. Furthermore, the joint analysis indicated that individuals in the decreasing low-stable sleep duration trajectory group who snored had the highest risk of cancer. The continuous monitoring of sleep duration may be of significant value in making prognoses in young individuals.

## Appendix

Multimedia Appendix 1 Supplementary information on methods and supplementary tables and figures.

## Abbreviations

CRP: c-reactive protein
CSHF: cause-specific hazard function
HR: hazard ratio
OSA: obstructive sleep apnea
PSQI: Pittsburgh Sleep Quality Index
REM: rapid eye movement
SDHF: subdistribution hazard function
TC: total cholesterol
TG: triglyceride
WC: waist circumference

## Data Availability

The data sets used and/or analyzed during this study are available from the corresponding author on reasonable request.
