# Supplementary material for: Age Differences in the Association of Sleep Duration Trajectory With Cancer Risk and Cancer-Specific Mortality: Prospective Cohort Study
Source: JMIR Public Health Surveill. 2024 Feb 7;10:e50836. doi: 10.2196/50836 (PMC10882471; doi:10.2196/50836)

**Supplementary Methods**

1.Pittsburgh Sleep Quality Index (PSQI) questionnaire

| Question | Score |
| --- | --- |
| Component 1: Subjective Sleep Quality: During the past month, how would you rate your sleep quality overall? | 0 = very good, 1 = fairly good, 2 = fairly bad, 3 = very bad |
| Component 2: Sleep Latency (Sleep Onset Latency): During the past month, how long (in minutes) has it usually taken you to fall asleep? | 0 = 15 minutes or less, 1 = 16-30 minutes, 2 = 31-60 minutes, 3 = More than 60 minutes |
| Component 3: Sleep Duration: During the past month, how many hours of actual sleep did you get at night? (Please exclude naps.) | 0 = 7-9 hours, 1 = 6 hours or less, 2 = More than 9 hours, 3 = Less than 6 hours |
| Component 4: Habitual Sleep Efficiency is calculated by dividing the number of hours slept by the number of hours spent in bed, multiplied by 100. | 0 = 85% or more, 1 = 75-84%, 2 = 65-74%, 3 = Less than 65% |
| Component 5: Sleep Disturbances: How often have you had trouble sleeping because you woke up in the middle of the night or early morning? | 0 = not during the past month, 1 = less than once a week, 2 = once or twice a week, 3 = three or more times a week |
| Component 6: Use of Sleep Medications: During the past month, how often have you taken medicine (prescribed or "over the counter") to help you sleep? | 0 = not during the past month, 1 = less than once a week, 2 = once or twice a week, 3 = three or more times a week |
| Component 7: Daytime Dysfunction: During the past month, how often have you had trouble staying awake while driving, eating meals, or engaging in social activity? | 0 = not during the past month, 1 = less than once a week, 2 = once or twice a week, 3 = three or more times a week |

2.The types of cancers included: digestive system cancers [esophageal cancer (C15), gastric cancer (C16), small intestine cancer (C17), colorectal cancer (C18-C21), liver cancer (C22.0), pancreatic cancer (C25), gallbladder and extrahepatic bile duct cancer (C23-C24)], lung cancer (C34), urinary system tumors [kidney cancer (C64-C65), bladder cancer (C67)], lymphoma (C81-C89) and leukemia (C90-C96), gender specific tumors [breast cancer (C50), cervix cancer (C53), uterus cancer (C54-C55), ovarian cancer (C56), prostate cancer (C61)], bone and soft tissue cancer (C40-C41) and other cancers.

3.In this study, we employed cause-specific hazard functions (CS) and subdistribution hazard functions (SD) to compute competing risk models. CS are statistical tools used in survival analysis to describe the occurrence rate of specific events while considering the influence of other events. This concept is typically applied in situations involving multiple competitive events where the event of interest may be subject to interference from other events. Cause-specific hazard functions aid in analyzing risk factors for different event types while accounting for the presence of other events. SD, on the other hand, are primarily used in competing risk analysis, where different event types may interact with each other. However, in certain cases, researchers are concerned with the probability of one event type occurring after another event type, without considering other events [1].

[1] Nie ZQ, Ou YQ, Qu YJ, Yuan HY, Liu XQ. Zhonghua Liu Xing Bing Xue Za Zhi. 2017;38(8):1127-1131. doi:10.3760/cma.j.issn.0254-6450.2017.08.026

**Table S1 Definition of covariates**

| **Covariates** | **Definition** |
| --- | --- |
| Sedentary | Maintain sitting position for at least 8 hours per day. |
| Regular physical activity | ≥ 3 times/week, ≥ 30 minutes/time of physical exercise is considered regular physical exercise |
| Smoke | Smoking was defined as having 1 cigarette/day at least for more than 6 months. |
| Alcohol use | Alcohol consumer was defined as having drunk ≥100 mL/day of alcohol lasting for more than 6 months, regardless of the type of alcohol. |
| Salt consumption | Salt consumption was self-reported and classified into three categories: low (<6 g/day), medium (6-9 g/day) or high (≥10 g/day). |
| Hypertension | Systolic blood pressure ≥ 140 mm Hg, and/or diastolic blood pressure ≥ 90 mm Hg, and/or previously diagnosed with hypertension. |
| Diabetes mellitus | Fasting blood glucose level ≥ 7.0mmoL/L, taking oral hypoglycemic drugs or insulin, or having a self-reported medical history. |
| Hepatitis B | Epidemiology, symptoms and positive hepatitis B surface antigen (HBsAg) are used to diagnose hepatitis B. The enzyme-linked immunosorbent assay was applied to detect HBsAg quantitatively with a standard operating procedure (Shanghai Kehua Bio-Engineering, KHB, Shanghai, China). |
| Liver cirrhosis, gallstones and polyps of the gallbladder | The ultrasonic examination was used to examine the abdominal region, including liver, gallbladder, pancreas and spleen of each participant after fasting for at least 8 hours by a panel of specialists. Liver cirrhosis, gallstone disease and gallbladder polyp were diagnosed by abdominal ultrasonography according to previous clinically established criteria [1,2] or through medical records from the Tangshan Medical Insurance System |
| Laboratory Testing | All participants underwent at least 8 hours of fasting and then received blood tests in the designated hospital, which were uniformly. All the serum samples were analyzed by an auto-analyzer (Hitachi 747; Hitachi, Tokyo, Japan) at the central laboratory of Kailuan General Hospital. |

[1] Saverymuttu SH, Joseph AE, Maxwell JD. Ultrasound scanning in the detection of hepatic fibrosis and steatosis. Br Med J (Clin Res Ed). 1986;292(6512):13-5.

[2] Esterson YB, Grimaldi GM. Radiologic Imaging in Nonalcoholic Fatty Liver Disease and Nonalcoholic Steatohepatitis. Clin Liver Dis. 2018;22(1):93-108.

**Table S2 The number of cancer cases with different sleep duration trajectories in all participants**

| Specific cancer site | Sleep duration trajectory patterns | | |  |
| --- | --- | --- | --- | --- |
|  | Normal-stable | Increasing median-stable | Decreasing low-stable | Overall |
| Lung cancer | 565 | 88 | 47 | 700 |
| Thyroid cancer | 82 | 7 | 5 | 94 |
| Breast cancer | 177 | 19 | 14 | 205 |
| Uterine/uterine cervix cancer | 57 | 9 | 5 | 71 |
| Lymphoma and leukemia | 30 | 4 | 1 | 35 |
| Kidney cancer | 47 | 4 | 2 | 53 |
| Bladder cancer | 74 | 13 | 3 | 90 |
| Prostate cancer | 46 | 2 | 4 | 52 |
| Pancreatic cancer | 44 | 7 | 5 | 56 |
| Head and neck cancer | 69 | 6 | 6 | 81 |
| Esophageal cancer | 76 | 8 | 7 | 91 |
| Stomach cancer | 143 | 26 | 14 | 183 |
| Colorectal cancer | 212 | 22 | 17 | 251 |
| Liver cancer | 150 | 21 | 5 | 176 |
| Gallbladder or extrahepatic bile duct cancer | 19 | 2 | 1 | 22 |
| Small intestine cancer | 21 | 2 | 0 | 23 |
| Bone and soft tissue cancer | 22 | 9 | 2 | 33 |
| Skin cancer | 19 | 7 | 1 | 27 |
| Other sites | 395 | 38 | 29 | 462 |

**Table S3 The number of cancer cases with different sleep duration trajectories in participants < 45 years**

| Specific cancer site | Sleep duration trajectory patterns | | |  |
| --- | --- | --- | --- | --- |
|  | Normal-stable | Increasing median-stable | Decreasing low-stable | Overall |
| Lung cancer | 86 | 8 | 9 | 103 |
| Thyroid cancer | 44 | 3 | 1 | 48 |
| Breast cancer | 46 | 3 | 0 | 49 |
| Uterine/uterine cervix cancer | 30 | 1 | 0 | 31 |
| Lymphoma and leukemia | 5 | 0 | 0 | 5 |
| Kidney cancer | 10 | 0 | 0 | 10 |
| Bladder cancer | 9 | 1 | 0 | 10 |
| Prostate cancer | 0 | 0 | 0 | 0 |
| Pancreatic cancer | 7 | 1 | 2 | 10 |
| Head and neck cancer | 28 | 2 | 2 | 32 |
| Esophageal cancer | 14 | 2 | 3 | 19 |
| Stomach cancer | 23 | 2 | 0 | 25 |
| Colorectal cancer | 36 | 2 | 4 | 42 |
| Liver cancer | 28 | 2 | 4 | 34 |
| Gallbladder or extrahepatic bile duct cancer | 1 | 1 | 0 | 2 |
| Small intestine cancer | 4 | 1 | 0 | 5 |
| Bone and soft tissue cancer | 6 | 1 | 0 | 7 |
| Skin cancer | 4 | 0 | 0 | 4 |
| Other sites | 71 | 6 | 5 | 82 |

**Table S4 The number of cancer cases with different sleep duration trajectories in participants ≥ 45 years**

| Specific cancer site | Sleep duration trajectory patterns | | |  |
| --- | --- | --- | --- | --- |
|  | Normal-stable | Increasing median-stable | Decreasing low-stable | Overall |
| Lung cancer | 479 | 80 | 38 | 597 |
| Thyroid cancer | 38 | 4 | 4 | 46 |
| Breast cancer | 236 | 16 | 14 | 156 |
| Uterine/uterine cervix cancer | 27 | 8 | 5 | 40 |
| Lymphoma and leukemia | 25 | 4 | 1 | 30 |
| Kidney cancer | 37 | 4 | 2 | 43 |
| Bladder cancer | 65 | 12 | 3 | 80 |
| Prostate cancer | 46 | 2 | 4 | 52 |
| Pancreatic cancer | 37 | 6 | 3 | 46 |
| Head and neck cancer | 41 | 4 | 4 | 49 |
| Esophageal cancer | 62 | 6 | 4 | 72 |
| Stomach cancer | 120 | 24 | 14 | 158 |
| Colorectal cancer | 176 | 20 | 13 | 209 |
| Liver cancer | 122 | 19 | 1 | 142 |
| Gallbladder or extrahepatic bile duct cancer | 18 | 1 | 1 | 20 |
| Small intestine cancer | 17 | 1 | 0 | 18 |
| Bone and soft tissue cancer | 16 | 8 | 2 | 26 |
| Skin cancer | 15 | 7 | 1 | 23 |
| Other sites | 324 | 32 | 24 | 380 |

**Table S5 Basic characteristics of the participants < 45 years by sleep duration trajectory**

| Characteristics | level | Normal-stable | Increasing median-stable | Decreasing low-stable | P-value |
| --- | --- | --- | --- | --- | --- |
| N |  | 17674 | 1577 | 698 |  |
| Age (Year) |  | 36.98(6.38) | 38.36(6.02) | 38.44(5.89) | <0.001 |
| Sex (%) | Men | 12811(72.5) | 1397(88.6) | 593(85.0) | <0.001 |
| BMI (%, kg/m^2^) | <18.5 | 7721(43.7) | 634(40.2) | 278(39.8) | 0.022 |
|  | 18.5-23.9 | 6660(37.7) | 644(40.8) | 280(40.1) |  |
|  | ≥24 | 3293(18.6) | 299(19.0) | 140(20.1) |  |
| BMI (kg/m^2^) |  | 24.85(3.67) | 24.99(3.61) | 25.01(3.60) | 0.214 |
| Waist circumference (cm) |  | 84.0[77.0,90.0] | 84.7[78.0,91.0] | 85.0[80.0,90.0] | <0.001 |
| Marital status | Married | 16585(93.8) | 1461(92.6) | 658(94.3) | 0.146 |
| Education | College graduate or above | 6200(35.1) | 614(38.9) | 184(26.4) | <0.001 |
| Regular physical activity (%) | Yes | 1153(6.5) | 119(7.5) | 52(7.4) | 0.201 |
| Sedentary time (%) | >8h | 4894(27.7) | 623(39.5) | 174(24.9) | <0.001 |
| Smoke (%) | Yes | 5248(29.7) | 813(51.6) | 247(35.4) | <0.001 |
| Alcohol use (%) | Yes | 2463(13.9) | 455(28.9) | 144(20.6) | <0.001 |
| Hypertension (%) | Yes | 4521(25.6) | 395(25.0) | 189(27.1) | 0.590 |
| Diabetes mellitus (%) | Yes | 729(4.1) | 61(3.9) | 34(4.9) | 0.537 |
| Snore (%) | Yes | 5681(32.1) | 861(54.6) | 241(34.5) | <0.001 |
| Salt consumption (%) | <6 g/d | 1598(9.0) | 195(12.4) | 64(9.2) | <0.001 |
|  | 6-10 g/d | 14366(81.3) | 1085(68.8) | 566(81.1) |  |
|  | >10 g/d | 1705(9.6) | 296(18.8) | 68(9.7) |  |
| CRP (mg/L) |  | 0.60[0.20,1.42] | 0.58[0.20,1.30] | 0.61[0.25,1.50] | 0.164 |
| Total cholesterol (mmol/L) |  | 4.72[4.11,5.37] | 4.82[4.16,5.52] | 4.81[4.18,5.54] | <0.001 |
| Triglyceride (mmol/L) |  | 1.21[0.83,1.89] | 1.22[0.83,2.09] | 1.21[0.81,1.88] | 0.354 |
| Fatty liver (%) | Yes | 4902(27.7) | 496(31.5) | 235(33.7) | <0.001 |
| Family history of tumor (%) | Yes | 549(3.1) | 87(5.5) | 24(3.4) | <0.001 |
| Gallstone disease (%) | Yes | 207(1.2) | 13(0.8) | 10(1.4) | 0.363 |
| Cirrhosis (%) | Yes | 94(0.5) | 9(0.6) | 4(0.6) | 0.971 |
| Hepatitis B virus infection (%) | Yes | 562(3.2) | 55(3.5) | 25(3.6) | 0.688 |

**Table S6 Basic characteristics of the participants ≥45 years by sleep duration trajectory**

| Characteristics | level | Normal-stable | Increasing median-stable | Decreasing low-stable | P-value |
| --- | --- | --- | --- | --- | --- |
| N |  | 27170 | 4300 | 1854 |  |
| Age (Year) |  | 56.00(7.74) | 57.05(8.38) | 56.17(8.37) | <0.001 |
| Sex (%) | Men | 21512(79.2) | 3279(76.3) | 1317(71.0) | <0.001 |
| BMI (%, kg/m^2^) | <18.5 | 9834(36.2) | 1627(37.8) | 711(38.3) | 0.029 |
|  | 18.5-23.9 | 12079(44.5) | 1908(44.4) | 810(43.7) |  |
|  | ≥24 | 5257(19.3) | 765(17.8) | 333(18.0) |  |
| BMI (kg/m^2^) |  | 25.27(3.36) | 25.11(3.29) | 25.06(3.33) | 0.001 |
| Waist circumference (cm) |  | 88.0[81.0, 94.0] | 87.0[81.0, 94.0] | 87.0[82.0, 94.0] | 0.034 |
| Marital status | Married | 26073(96.0) | 4018(93.4) | 1781(96.1) | <0.001 |
| Education | College graduate or above | 3975(14.6) | 960(22.3) | 312(16.8) | <0.001 |
| Regular physical activity (%) | Yes | 4874(17.9) | 1261(29.3) | 327(17.6) | <0.001 |
| Sedentary time(%) | > 8h | 5937(21.9) | 1582(36.8) | 432(23.3) | <0.001 |
| Smoke (%) | Yes | 8006(29.5) | 1714(39.9) | 487(26.3) | <0.001 |
| Alcohol use (%) | Yes | 5016(18.5) | 1208(28.1) | 342(18.4) | <0.001 |
| Hypertension (%) | Yes | 13094(48.2) | 2000(46.5) | 803(43.3) | <0.001 |
| Diabetes mellitus (%) | Yes | 2841(10.5) | 441(10.3) | 192(10.4) | 0.919 |
| Snore (%) | Yes | 10157(37.4) | 2551(59.3) | 709(38.2) | <0.001 |
| Salt consumption (%) | <6 g/d | 2449(9.0) | 565(13.1) | 133(7.2) | <0.001 |
|  | 6-10 g/d | 21985(80.9) | 2965(69.0) | 1534(82.7) |  |
|  | >10 g/d | 2728(10.0) | 767(17.8) | 187(10.1) |  |
| CRP (mg/L) |  | 0.86[0.31, 2.45) | 0.90[0.38, 2.23] | 0.88[0.32, 2.30] | 0.452 |
| Total cholesterol (mmol/L) |  | 5.00[4.37, 5.67] | 5.10[4.42, 5.75] | 5.03[4.43, 5.69] | <0.001 |
| Triglyceride (mmol/L) |  | 1.32[0.94, 1.97] | 1.30[0.91, 1.95] | 1.30[0.89, 1.92] | 0.017 |
| Fatty liver (%) | Yes | 9610(35.4) | 1701(39.6) | 672(36.2) | <0.001 |
| Family history of tumor (%) | Yes | 1173(4.3) | 350(8.1) | 66(3.6) | <0.001 |
| Gallstone disease (%) | Yes | 771(2.8) | 133(3.1) | 49(2.6) | 0.548 |
| Cirrhosis (%) | Yes | 243(0.9) | 38(0.9) | 20(1.1) | 0.712 |
| Hepatitis B virus infection (%) | Yes | 650(2.4) | 96(2.2) | 49(2.6) | 0.617 |

**Table S7 Bonferroni correction**

| P | Normal-stable VS Increasing median-stable | Increasing median-stable VS Decreasing low-stable | Normal-stable VS Decreasing low-stable |
| --- | --- | --- | --- |
| Age (Year) | <0.001 | 0.008 | <0.001 |
| Age (%) | <0.001 | 0.642 | <0.001 |
| Sex (%) | <0.001 | <0.001 | 0.052 |
| BMI (%) | 0.040 | 0.808 | 0.623 |
| BMI (kg/m2) | 0.527 | 0.730 | 0.412 |
| Waist circumference (cm) | 0.002 | 0.086 | <0.001 |
| Marital status (%) | <0.001 | <0.001 | 0.330 |
| Education (%) | <0.001 | <0.001 | <0.001 |
| Sedentary time (%) | <0.001 | <0.001 | 0.658 |
| Regular physical activity (%) | <0.001 | <0.001 | 0.046 |
| Smoke (%) | <0.001 | <0.001 | 0.405 |
| Alcohol use (%) | <0.001 | <0.001 | 0.002 |
| Hypertension (%) | 0.031 | 0.111 | 0.696 |
| Diabetes mellitus (%) | 0.130 | 0.668 | 0.114 |
| Snore (%) | <0.001 | <0.001 | 0.053 |
| Salt consumption (%) | <0.001 | <0.001 | 0.079 |
| Family history of tumor (%) | <0.001 | <0.001 | 0.453 |
| hs-CRP (mg/L) | 0.001 | 0.713 | 0.006 |
| Total cholesterol (mmol/L) | <0.001 | 0.413 | <0.001 |
| Triglyceride (mmol/L) | 0.828 | 0.296 | 0.296 |
| Fatty liver (%) | <0.001 | 0.113 | 0.001 |
| Gallstone disease (%) | 0.150 | 0.693 | 0.711 |
| Cirrhosis (%) | 0.748 | 0.603 | 0.342 |
| Hepatitis B virus infection (%) | 0.581 | 0.429 | 0.594 |

*P < 0.017 is considered to indicate a statistical difference.

**Table S8 Hazard ratios (HRs) for the association between sleep duration trajectory patterns and specific site cancer risk in all participants**

| **Specific site cancer** | **Sleep duration trajectory patterns ^b^** | | |
| --- | --- | --- | --- |
|  | Normal-stable | Increasing median-stable | Decreasing low-stable |
| Lung cancer | Ref. | 1.11(0.86,1.43) | **1.51(1.08,2.12)** |
| Thyroid cancer | Ref. | 0.79(0.34,1.86) | 1.25(0.45,3.43) |
| Breast cancer a | Ref. | 0.83(0.48,1.41) | 1.19(0.64,2.24) |
| Uterine/uterine cervix cancer a | Ref. | 1.32(0.58,2.99) | 1.46(0.51,4.19) |
| Lymphoma and leukemia | Ref. | 1.28(0.61,2.69) | 1.83(0.68,4.87) |
| Kidney cancer | Ref. | 0.60(0.19,1.83) | 0.67(0.14,3.11) |
| Bladder cancer | Ref. | 1.10(0.56,2.15) | 0.66(0.19,2.27) |
| Prostate cancer a | Ref. | 0.33(0.07,1.42) | 1.64(0.50,5.34) |
| Pancreatic cancer | Ref. | 1.58(0.64,3.87) | **3.08(1.05,9.08)** |
| Head and neck cancer | Ref. | 0.62(0.25,1.54) | 1.58(0.60,4.15) |
| Esophageal cancer **^c^** | Ref. | 0.65(0.29,1.42) | 1.48(0.62,3.61) |
| Stomach cancer **^c^** | Ref. | 1.51(0.93,2.41) | **2.06(1.10,3.87)** |
| Colorectal cancer **^c^** | Ref. | 0.83(0.51,1.34) | 1.70(0.97,2.26) |
| Liver cancer **^c^** | Ref. | 1.06(0.64,1.77) | 0.65(0.25,1.67) |
| Gallbladder or extrahepatic bile duct cancer **^c^** | Ref. | 0.82(0.16,4.11) | 1.36(0.15,8.29) |
| Digestive system cancers **^c^** | Ref. | 1.03(0.81,1.32) | **1.50(1.08,2.07)** |
| Small intestine cancer **^c^** | Ref. | 0.72(0.15,3.52) | NA |
| Bone and soft tissue cancer | Ref. | **2.80(1.11,6.05)** | 1.81(0.36,7.08) |

^a^ Analyses were only performed in men or women.

^b^ The model was adjusted for age (continuous), sex (categorical), BMI (continuous), WC (continuous), marital status (categorical), education level (categorical), sedentary time (categorical), physical activity (categorical), smoking (categorical), alcohol use (categorical), hypertension (categorical), diabetes (categorical), snoring (categorical), salt intake (categorical), family history of tumor (categorical), CRP (continuous), TC (continuous), TG (continuous) and sleep duration in 2010 (continuous).

^c^ Further adjusted for HBV, liver cirrhosis, fatty liver disease, gallstone disease and gallbladder polyp.

Bold indicates statistically significant differences.

**Table S9 Subgroup analyses for the association between sleep duration trajectory and cancer risk**

| **Snoring (n=6783)** | N | Normal-stable | Increasing median-stable | Decreasing low-stable | **Without snoring (n=13166)** | N | Normal-stable | Increasing median-stable | Decreasing low-stable |
| --- | --- | --- | --- | --- | --- | --- | --- | --- | --- |
| Lung cancer | 37 | Ref. | 1.70(0.68,4.28) | **3.24(1.10,10.72)** | Lung cancer | 66 | Ref. | 0.33(0.04,2.48) | **2.32(1.36,9.93)** |
| Esophageal cancer ^a^ | 8 | Ref. | NA | 2.15(0.66,12.49) | Esophageal cancer ^a^ | 11 | Ref. | 3.52(0.62,17.20) | 7.91(0.90,27.20) |
| Pancreatic cancer ^a^ | 5 | Ref. | 2.26(0.25,15.24) | **9.09(1.23,58.26)** | Pancreatic cancer ^a^ | 5 | Ref. | NA | NA |
| Liver cancer ^a^ | 14 | Ref. | 0.62(0.08,5.71) | **8.06(2.18,21.33)** | Liver cancer ^a^ | 19 | Ref. | 0.72(0.10,6.03) | 0.99(0.10,9.47) |
| Digestive system cancers ^a^ | 53 | Ref. | 0.68(0.26,1.82) | **3.05(1.09,8.55)** | Digestive system cancers ^a^ | 84 | Ref. | 1.14（0.47,2.78） | 2.04(0.82,5.07) |
| Overall cancer risk | 157 | Ref. | 0.87(0.56,1.51) | **2.40(1.23,4.67)** | Overall cancer risk | 346 | Ref. | 1.12(0.67,1.86) | **1.84(1.06,3.16)** |
| P for interaction=0.173 | | | | | | | | | |
| **Men (n=14801)** | N | Normal-stable | Increasing median-stable | Decreasing low-stable | **Women (n=5148)** | N | Normal-stable | Increasing median-stable | Decreasing low-stable |
| Lung cancer | 82 | Ref. | 1.11(0.48,2.55) | **2.23(1.32,6.91)** | Lung cancer | 21 | Ref. | NA | **8.01(1.38,26.60)** |
| Esophageal cancer ^a^ | 19 | Ref. | 1.18(0.24,5.82) | **5.24(1.13,14.16)** | Esophageal cancer ^a^ | 0 | Ref. | NA | NA |
| Pancreatic cancer ^a^ | 8 | Ref. | 1.31(0.13,11.95) | 4.30(0.34,34.19) | Pancreatic cancer ^a^ | 2 | Ref. | NA | NA |
| Liver cancer ^a^ | 32 | Ref. | 0.74(0.16,3.34) | **4.46(1.26,14.79)** | Liver cancer ^a^ | 1 | Ref. | NA | NA |
| Digestive system cancers ^a^ | 120 | Ref. | 0.97(0.50,1.91) | **2.27(1.06,4.68)** | Digestive system cancers ^a^ | 17 | Ref. | NA | **5.83(1.13,30.03)** |
| Overall cancer risk | 321 | Ref. | 0.99(0.65,1.52) | **2.07(1.26,3.40)** | Overall cancer risk | 182 | Ref. | 0.53(0.16,1.71) | **2.45(1.11,5.38)** |
| P for interaction=0.743 | | | | | | | | | |
| **BMI<24 (n=8607)** | N | Normal-stable | Increasing median-stable | Decreasing low-stable | **BMI≥24 (n=11342)** | N | Normal-stable | Increasing median-stable | Decreasing low-stable |
| Lung cancer | 43 | Ref. | 1.02(0.29,3.62) | **3.37(1.01,11.06)** | Lung cancer | 60 | Ref. | 1.21(0.45,3.27) | **3.68(1.27,6.28)** |
| Esophageal cancer ^a^ | 10 | Ref. | 0.99(0.13,7.83) | **7.79(2.02,30.16)** | Esophageal cancer ^a^ | 9 | Ref. | 1.76(0.22,10.09) | NA |
| Pancreatic cancer ^a^ | 4 | Ref. | NA | **5.59(3.26,96.37)** | Pancreatic cancer ^a^ | 6 | Ref. | 2.26(0.20,25.20) | 4.92(0.30,61.11) |
| Liver cancer ^a^ | 15 | Ref. | 0.81(0.10,7.12) | NA | Liver cancer ^a^ | 18 | Ref. | 0.57(0.07,4.69) | **7.16(1.72,29.77)** |
| Digestive system cancers ^a^ | 60 | Ref. | 0.54(0.16,1.84) | 2.66(0.97,7.29) | Digestive system cancers ^a^ | 77 | Ref. | 1.10(0.50,2.44) | **2.26(0.89,5.74)** |
| Overall cancer risk | 231 | Ref. | 0.90(0.50,1.64) | **2.79(1.58,5.02)** | Overall cancer risk | 272 | Ref. | 1.05(0.65,1.68) | 1.58(0.87,2.84) |
| P for interaction=0.206 | | | | | | | | | |

The model was adjusted for age (continuous), sex (categorical), BMI (continuous), WC (continuous), marital status (categorical), education level (categorical), sedentary time (categorical), physical activity (categorical), smoking (categorical), alcohol use (categorical), hypertension (categorical), diabetes (categorical), snoring (categorical), salt intake (categorical), family history of tumor (categorical), CRP (continuous), TC (continuous), TG (continuous) and sleep duration in 2010 (continuous).

^a^ Further adjusted for HBV, liver cirrhosis, fatty liver disease, gallstone disease and gallbladder polyp.

Abbreviation, RPA, regular physical activity

Bold indicates statistically significant differences

**Table S10 Joint analysis for snoring and sleep duration trajectory.**

|  | G1 | G2 | G3 | G4 | G5 | G6 |
| --- | --- | --- | --- | --- | --- | --- |
| Lung cancer | Ref. | 0.30(0.04,2.23) | **3.09(1.14,6.41)** | 0.91(0.56,1.49) | 1.79(0.77,4.22) | **4.29(1.40,9.11)** |
| Esophageal cancer ^a^ | Ref. | 3.09(0.58,15.57) | **6.71(1.11,27.69)** | 1.71(0.57,5.11) | NA | **5.45(1.53,15.28)** |
| Pancreatic cancer ^a^ | Ref. | NA | NA | 1.34(0.26,6.76) | 3.78(0.32,23.61) | **9.55(1.23,62.12)** |
| Liver cancer ^a^ | Ref. | 0.86(0.11,6.82) | 1.59(0.19,10.44) | 1.34(0.59,3.03) | 0.72(0.09,5.81) | **8.96(2.07,18.82)** |
| Digestive system cancers ^a^ | Ref. | 1.10(0.46,2.62) | **2.22(1.04,5.21)** | 1.08(0.72,1.64) | 0.71(0.28,1.64) | **2.98(1.17,7.56)** |
| Overall cancer risk | Ref. | 1.06(0.64,1.74) | **1.70(1.01,2.87)** | 0.92(0.74,1.15) | 0.86(0.51,1.44) | **2.62(1.46,4.70)** |

G1, group 1, participants in normal-stable group but without snoring.

G2, group 2, participants in increasing median-stable group but without snoring.

G3, group 3, participants in decreasing low-stable group but without snoring.

G4, group 4, participants in normal-stable group with snoring.

G5, group 5, participants in increasing median-stable group with snoring.

G6, group 6, participants in decreasing low-stable group with snoring.

The model was adjusted for age (continuous), sex (categorical), BMI (continuous), WC (continuous), marital status (categorical), education level (categorical), sedentary time (categorical), physical activity (categorical), smoking (categorical), alcohol use (categorical), hypertension (categorical), diabetes (categorical), snoring (categorical), salt intake (categorical), family history of tumor (categorical), CRP (continuous), TC (continuous), TG (continuous) and sleep duration in 2010 (continuous).

Bold indicates statistically significant difference.

**Table S11 Competing risk model for the association between sleep duration trajectory and cancer risk**

| **Specific cancer site** | **Sleep duration trajectory patterns ^a^** | | |
| --- | --- | --- | --- |
|  | **Normal stable** | **Increasing median-stable** | **Decreasing low-stable** |
| **SD models** |  |  |  |
| Lung cancer | Ref. | 1.10(0.51,2.01) | **2.23(1.17,5.25)** |
| Esophageal cancer ^b^ | Ref. | 1.01(0.20,4.76) | **4.05(1.13,15.03)** |
| Pancreatic cancer ^b^ | Ref. | 1.30(0.16,8.97) | **6.88(1.38,27.69)** |
| Liver cancer ^b^ | Ref. | 0.60(0.13,2.72) | **3.08(1.08,10.40)** |
| Digestive system cancers ^b^ | Ref. | 0.79(0.41,1.55) | **1.99(1.23,4.97)** |
| Overall cancer risk | Ref. | 0.92(0.64,1.34) | **1.97(1.12,3.08)** |
| **CS models** |  |  |  |
| Lung cancer | Ref. | 1.13(0.56,2.54) | **2.42(1.07,5.53)** |
| Esophageal cancer ^b^ | Ref. | 1(0.23,4.97) | **4.07(1.12,14.89)** |
| Pancreatic cancer ^b^ | Ref. | 1.32(0.19,12.38) | **7(1.40,42.31)** |
| Liver cancer ^b^ | Ref. | 0.67(0.21,3.03) | **3.37(1.19,9.66)** |
| Digestive system cancers ^b^ | Ref. | 0.82(0.43,1.63) | **1.93(1.05,3.62)** |
| Overall cancer risk | Ref. | 0.4(0.67,1.43) | **2.01(1.39,3.17)** |

^a^ The model was adjusted for age (continuous), sex (categorical), BMI (continuous), WC (continuous), marital status (categorical), education level (categorical), sedentary time (categorical), physical activity (categorical), smoking (categorical), alcohol use (categorical), hypertension (categorical), diabetes (categorical), snoring (categorical), salt intake (categorical), family history of tumor (categorical), CRP (continuous), TC (continuous), TG (continuous) and sleep duration in 2010 (continuous).

^b^ Further adjusted for HBV, liver cirrhosis, fatty liver disease, gallstone disease and gallbladder polyp.

Bold indicates statistically significant difference.

**Table S12 Sensitivity analyses for sleep duration trajectory and cancer risk**

| **Specific cancer site** | **Sleep duration trajectory patterns** | | |
| --- | --- | --- | --- |
|  | **Normal-stable** | **Increasing median-stable** | **Decreasing low-stable** |
| **Excluding participants with a family history of tumor(n=19289) ^a^** | | | |
| Lung cancer | Ref. | 1.14(0.52,2.47) | **3.63(1.64,7.07)** |
| Esophageal cancer ^b^ | Ref. | 1.11(0.23,5.41) | **4.91(1.07,19.58)** |
| Pancreatic cancer ^b^ | Ref. | 1.77(0.19,15.10) | **10.64(1.62,43.44)** |
| Liver cancer ^b^ | Ref. | 0.64(0.14,2.88) | **3.68(1.04,10.01)** |
| Digestive system cancers ^b^ | Ref. | 0.86(0.44,1.66) | **2.44(1.23,4.84)** |
| Overall cancer risk | Ref. | 0.99(0.69,1.44) | **2.07(1.36,3.13)** |
| **Excluding participants with hepatitis B virus infection (n=19307) ^a^** | | | |
| Lung cancer | Ref. | 1.27(0.58,2.80) | **3.67(1.58,7.54)** |
| Esophageal cancer ^b^ | Ref. | 1.15(0.23,5.67) | **4.82(1.02,19.71)** |
| Pancreatic cancer ^b^ | Ref. | 1.82(0.19,12.55) | **1.33(1.65,40.66)** |
| Liver cancer ^b^ | Ref. | 0.55(0.07,4.58) | 3.86(0.67,20.44) |
| Digestive system cancers ^b^ | Ref. | 0.88(0.44,1.77) | **2.34(1.11,4.94)** |
| Overall cancer risk | Ref. | 1.05(0.72,1.54) | **1.89(1.21,2.96)** |
| **Excluding participants with regular physical activity (n=18625)** | | | |
| Lung cancer | Ref. | 0.97(0.43,2.23) | **3.44(1.54,6.68)** |
| Esophageal cancer ^b^ | Ref. | 1.40(0.28,6.95) | **6.64(1.44,20.73)** |
| Pancreatic cancer ^b^ | Ref. | 1.78(0.19,11.13) | **11.67(1.63,43.62)** |
| Liver cancer ^b^ | Ref. | 0.68(0.15,3.09) | **3.85(1.07,12.80)** |
| Digestive system cancers ^b^ | Ref. | 0.95(0.49,1.86) | **2.71(1.36,5.42)** |
| Overall cancer risk | Ref. | 0.91(0.61,1.35) | **2.10(1.37,3.22)** |
| **Exclude participants with cancer diagnosed within 1st year of follow-up (n=19883) ^a^** | | | |
| Lung cancer | Ref. | 1.17(0.53,2.57) | **3.12(1.35,7.38)** |
| Esophageal cancer ^b^ | Ref. | 1.17(0.24,5.79) | **4.91(1.04,16.06)** |
| Pancreatic cancer ^b^ | Ref. | 1.88(0.20,16.64) | 5.46(0.45,37.12) |
| Liver cancer ^b^ | Ref. | 0.73(0.16,3.36) | **4.39(1.23,8.70)** |
| Digestive system cancers ^b^ | Ref. | 0.82(0.41,1.64) | **2.37(1.17,4.80)** |
| Overall cancer risk | Ref. | 1.02(0.70,1.50) | **2.03(1.31,3.11)** |
| **Adjusting for time-varying covariates ^c^** | | | |
| Lung cancer | Ref. | 0.97(0.45,1.95) | **2.35(1.14,4.87)** |
| Esophageal cancer ^b^ | Ref. | 1.57(0.30,4.89) | **4.39(1.33,15.07)** |
| Pancreatic cancer ^b^ | Ref. | 1.34(0.14,12.15) | **7.27(1.57,30.24)** |
| Liver cancer ^b^ | Ref. | 0.57(0.19,1.90) | **3.14(1.10,9.05)** |
| Digestive system cancers ^b^ | Ref. | 0.78(0.41,1.54) | **2.27(1.224.20)** |
| Overall cancer risk | Ref. | 0.86(0.57,1.34) | **1.72(1.16,2.73)** |
| **Excluding participants taking sleep medication at least once during the past month (n=19741)** | | | |
| Lung cancer | Ref. | 1.14(0.52,2.49) | **3.62(1.63,8.06)** |
| Esophageal cancer ^b^ | Ref. | 1.11(0.23,5.43) | **4.89(1.06,12.51)** |
| Pancreatic cancer ^b^ | Ref. | 2.22(0.22,12.27) | **12.32(1.73,42.68)** |
| Liver cancer ^b^ | Ref. | 0.64(0.14,2.89) | **3.68(1.04,12.96)** |
| Digestive system cancers ^b^ | Ref. | 0.85(0.44,1.66) | **2.42(1.22,4.80)** |
| Overall cancer risk | Ref. | 1(0.69,1.45) | **2.02(1.33,3.08)** |
| **Adjusting for other components in PSQI ^d^** | | | |
| Lung cancer | Ref. | 1.13(0.52,2.47) | **3.64(1.64,8.09)** |
| Esophageal cancer ^b^ | Ref. | 1.10(0.23,5.40) | **4.87(1.06,18.46)** |
| Pancreatic cancer ^b^ | Ref. | 1.80(0.19,15.35) | **11.70(1.64,43.46)** |
| Liver cancer ^b^ | Ref. | 0.65(0.14,2.94) | **3.68(1.04,13.02)** |
| Digestive system cancers ^b^ | Ref. | 0.85(0.44,1.64) | **2.42(1.22,4.79)** |
| Overall cancer risk | Ref. | 0.99(0.68,1.43) | **2.06(1.36,3.12)** |

^a^ The model was adjusted for age (continuous), sex (categorical), BMI (continuous), WC (continuous), marital status (categorical), education level (categorical), sedentary time (categorical), physical activity (categorical), smoking (categorical), alcohol use (categorical), hypertension (categorical), diabetes (categorical), snoring (categorical), salt intake (categorical), family history of tumor (categorical), CRP (continuous), TC (continuous), TG (continuous) and sleep duration in 2010 (continuous).

^b^ Further adjusted for HBV, liver cirrhosis, fatty liver disease, gallstone disease and gallbladder polyp.

^c^ The model was adjusted for age (time-varying), sex (time-varying), BMI (time-varying), WC (time-varying), marital status (time-varying), education level (time-varying), sedentary time (time-varying), physical activity (time-varying), smoking (time-varying), alcohol use (time-varying), hypertension (time-varying), diabetes (time-varying), snoring (time-varying), salt intake (time-varying), family history of tumor (time-varying), CRP (time-varying), TC (time-varying), TG (time-varying).

^d^ The model was adjusted for age (continuous), sex (categorical), BMI (continuous), WC (continuous), marital status (categorical), education level (categorical), sedentary time (categorical), physical activity (categorical), smoking (categorical), alcohol use (categorical), hypertension (categorical), diabetes (categorical), snoring (categorical), salt intake (categorical), family history of tumor (categorical), CRP (continuous), TC (continuous), TG (continuous), sleep duration in 2010 (continuous), daytime dysfunction and use of sleep medication.

Bold indicates statistically significant difference.

Figure S1 Hazard ratios for the association between sleep duration trajectory patterns and specific site cancer risk in participants of different ages.


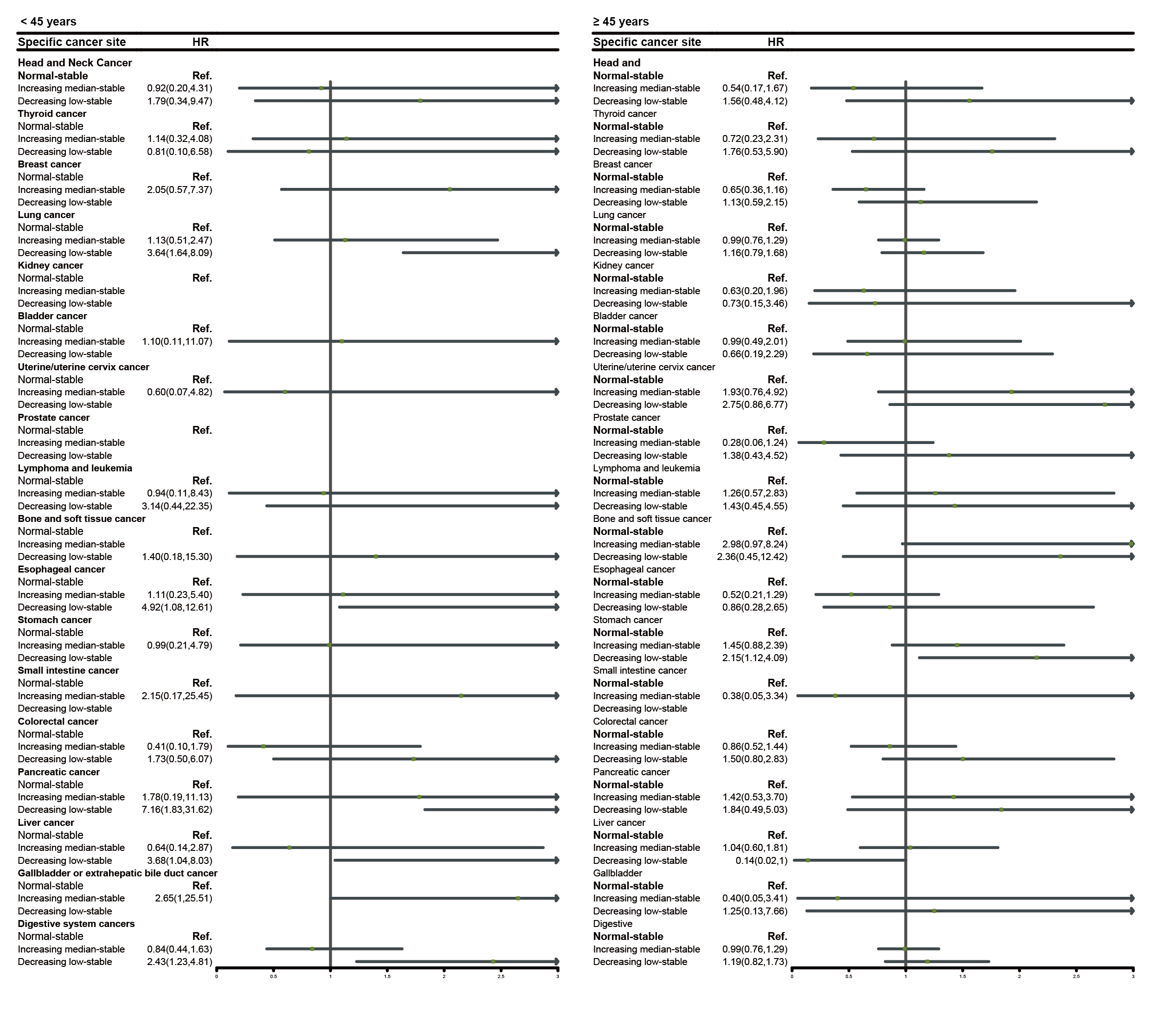

Supplement: Multimedia Appendix 1 [file publichealth_v10i1e50836_app1.docx]
